# Laparoscopic repair of a Morgagni hernia

**DOI:** 10.4103/0972-9941.16532

**Published:** 2005-06

**Authors:** J. M. Sherigar, A. D. Dalal, J. R. Patel

**Affiliations:** Department of General Surgery, Sheth V.S. General Hospital, Sheth K.M. School of Postgraduate Medicine & Research, N.H.L. Muni. Medical College, Ahmedabad, India

**Keywords:** Diaphragmatic hernia, Laparoscopy, Morgagni hernia

## Abstract

We report a case of laparoscopic repair of symptomatic Morgagni hernia (MH) in an adult. A tension-free closure of the defect was carried out using a polypropylene mesh. The recovery was quick and uneventful. Two years after surgery, the patient is doing well. A search of the English-language surgical literature revealed a total of 55 cases of laparoscopic repair of MH reported: 40 in adults and 15 in children. The various modalities of diagnosis, operative techniques, and disease presentation are discussed.

## INTRODUCTION

Morgagni hernia (MH) is a congenital hernia occurring in the retroxiphoid region and accounts for approximately 1–3%[1] of surgically treated diaphragmatic hernias. The traditional treatment of MH has involved an open surgical repair either by the transabdominal or transthoracic route. With the advances in laparoscopic technique, many cases of MH have been treated successfully using this approach. We describe one such case and review the literature on the subject.

## CASE REPORT

**Figure 1:**
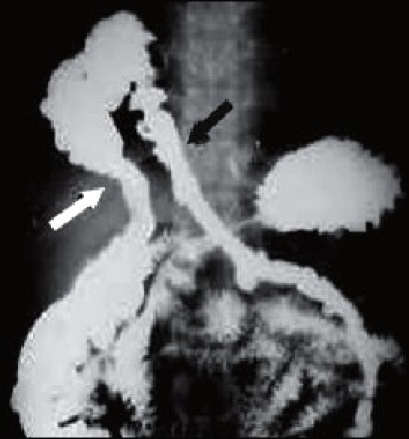
Contrast study revealing herniation of transverse colon

A 32-year-old man presented with a 2-year history of right upper abdominal and epigastric pain unrelated to intake of food. The pain was relieved by passing stool or flatus. The patient was well built and abdominal examination was unremarkable. Examination of the respiratory system revealed reduced air entry at right lower zone and the liver dullness was impaired on percussion. The biochemical and hematological tests were within normal limits. Posteroanterior and lateral radiographs of the chest showed localized elevation of right dome of the diaphragm near the cardiophrenic angle with gas shadows within the chest. Ultrasonography of abdomen revealed a 6 × 5 cm defect in the right dome of the diaphragm and bowel loops were seen on the superior aspect of the liver. Herniation of transverse colon in the right side of the chest was confirmed by contrast study [Figure 1].

An elective laparoscopic mesh repair was planned under general anesthesia. Patient was placed in supine position. The surgeon stood on the left side with the assistant on the opposite side. A 10-mm umbilical port for an 0° telescope was established and two other ports were placed – a 10-mm one in the epigastrium and a 5-mm port in the right lumbar region in the midclavicular line. Examination of the upper abdomen showed presence of a defect on the right side behind the sternum in the anterior aspect of the diaphragm. This contained the omentum, the transverse colon and part of the liver. Contents of the hernia were gently reduced. Adhesions at the edges of the defect were suture ligated and excised. No attempt was made to excise the sac. A 7.5 × 7.5 cm polypropylene mesh sutured in place with a monofilament polypropylene suture was used to close the defect. An over and over continuous suture taking the full thickness of the edge of the defect anchored the mesh. Sheath at umbilical port was sutured with 1/0 polyglactin suture and the port sites were infiltrated with 0.25% bupivacaine. The operation lasted 120 min. Postoperative period was uneventful and the patient was discharged on third postoperative day. At a follow up of 2 years the patient remains asymptomatic.

## DISCUSSION

Morgagni hernia is a diaphragmatic hernia occurring through a congenital defect in the retroxiphoid region. The diaphragmatic defect described by both Morgagni and Larrey is a triangular space between the muscle fibers of the diaphragm that originates from the xiphisternum and the costal margin and inserts on to the central tendon of the diaphragm. This space is referred to as the foramen of Morgagni or the space of Larrey. Although it is more common on the right, in rare instances the hernia can be bilateral.

Majority of the patients with MH are asymptomatic and diagnosis is usually made from routine chest X-ray. Some patients may present with nonspecific cardiac, respiratory, or gastrointestinal symptoms. Rarely, acute abdominal or thoracic symptoms caused by obstruction and strangulation of the bowel may lead to the presentation.[2] Children commonly present with repeated episodes of chest infections; rarely MH may manifest in neonates as acute respiratory distress syndrome. The organs most commonly seen in the hernial sac (in order of decreasing frequency) are the omentum, the colon, the stomach, the liver, and the small intestine. Confirmation of the diagnosis is by computerized tomography, contrast study, magnetic resonance imaging, or laparoscopy. Laparoscopy provides an excellent modality both for diagnosis as well as for repair of a MH. While uncommon, the possibility of incarceration and strangulation of viscera is likely in all cases diagnosed to have a MH and therefore surgical repair is recommended.[3][4]

Traditionally the surgical management has involved an open transthoracic or transabdominal repair with suturing of the edge of the diaphragm to the retrosternal and retrocostal endothoracic fascia and/or posterior rectus sheath. These approaches can involve significant morbidity. In recent years with the advent of laparoscopic surgery, several cases of laparoscopic repair have been reported in the surgical literature both in adult and children. Hussong et al.[5] reported a repair through video assisted thoracic surgery. The first laparoscopic surgery was reported by Kuster et al. in 1992.[1] They repaired the defect with a percutaneously introduced monofilament suture tied in an over and over manner. The surgical literature till date carries 55 reported cases of minimally invasive repair of MH: 40 in adults and 15 in children.

Due to the relative rarity of condition, there is no consensus as to the optimum laparoscopic technique and various approaches have been reported. Most reports describe the use of a mesh fixed in place with sutures or staples of different variety. Around half the reports describe the use of a polypropylene mesh and others have used intermittent or continuous nonabsorbable suture to close the defect. Orita et al.[6] reported laparoscopic repair under an abdominal wall lift technique. He approximated the defect with sutures and reinforced it with a mesh. Chang et al.[2] used a laparoscopic suturing device to close the defect. Direct suturing technique violates the concept of a tension-free repair. In general, smaller defects can be closed with sutures without tension, and a mesh is used to bridge larger defects. We used polypropylene mesh and sutured it securely with a continuous intracorporeal suture. We believe that a mesh repair provides tension-free closure and is therefore superior to a sutured repair. Permeability of the mesh allows the seroma collecting in the residual sac to drain out into peritoneal cavity.[7] Reperitonealization is generally not required as the liver covers the mesh and keeps it away from the loops of bowel. If needed, the mesh may be covered with omentum to avoid adhering of intra-abdominal viscera. The reported cases do not allow one to draw a conclusion regarding a clear superiority of one type of mesh over any other. Filipi et al.[8] used e-PTFE (Goretex) mesh (W L Gore and Associates, Flagstaff, USA) for the repair and Blazquez et al.[9] closed the defect with a Parietex bilayered composite mesh (Sofradim Corporation, Wrentham, USA). This mesh has been claimed to protect the viscera from direct contact with the mesh during the process of integration.

The issue of removal of the hernial sac during repair of a MH is controversial. In over half the reported cases the sac was not removed. When the sac is small it can be excised completely. Many authors prefer to leave the sac *in situ* especially when it is adherent to the pleura or pericardium to avoid the risk of pneumothorax or pneumopericardium. There is no available literature to recommend whether this influences the recurrence or formation of a cyst. In our case we did not attempt to remove the sac.

Indications for the laparoscopic treatment in children are delayed presentation of the MH and a symptom-free interval and no or minimal respiratory symptoms. On the other hand, in neonates with respiratory distress MH should not be treated laparoscopically because of the risks associated with CO_2_ pneumoperitoneum.[10] As in adults the best method of closure of the defect in children remains debatable. The defect may be closed either by suture closure, placement of a mesh or combination of both. From the surgical literature the most common method used during laparoscopic repair of MH in children appears to be suture closure. As regards excision of sac, controversy exists as in adults. Limited experience found that laparoscopic approach to be an effective surgical technique preferred to conventional laparotomy in selected pediatric patients.

## CONCLUSION

Surgical correction forms the mainstay of treatment of MH. Laparoscopy can be diagnostic as well as therapeutic. Conventional open repair includes hernia reduction, resection of the hernial sac, whenever possible, and suture closure of the defect. The same can be easily and safely performed by laparoscopy. Laparoscopy provides excellent access to the diaphragmatic region, is associated with minimal wound complications, carries an aesthetic benefit and also results in less postoperative pain and a faster recovery. It may be difficult to advocate any one technique of minimally invasive repair over the other based on the reported literature as no major complications or recurrences have been reported with the different methods of laparoscopic repair. We believe that the mesh repair with suture fixation is secure and satisfactory.
